# Habitat requirements of riparian arthropods on gravel bars: Implications for conservation and management of braided river floodplains

**DOI:** 10.1371/journal.pone.0274977

**Published:** 2022-09-27

**Authors:** Reena Wessels, Andrea Sundermann

**Affiliations:** 1 Department for Animal Ecology, Faculty of Biology, Philipps-Universität Marburg, Marburg, Germany; 2 Department of River Ecology and Conservation, Senckenberg Research Institute and Natural History Museum Frankfurt, Gelnhausen, Germany; 3 Department Aquatic Ecotoxicology, Institute of Ecology, Evolution and Diversity, Goethe University, Frankfurt am Main, Germany; Universite de Pau et des Pays de l’Adour College STEE Sciences et Technologies pour l’Energie et l’Environnement, FRANCE

## Abstract

In their pristine state, river landscapes consist of complex mosaics of aquatic and terrestrial habitats. They are highly dynamic and, with their harsh environments, offer living space for many specialists. In the present study, the habitat choice of specialists of the riparian arthropod community was studied on a near-natural stretch of the Upper Isar River. Study period was between May and July 2011. Araneae, Formicidae and Staphylinidae were the most common taxa. The dominant species was *Pardosa wagleri* with 1,092 individuals, followed by *Arctosa cinerea* with 184 and *Paederidus rubrothoracicus* with 154 individuals. These three species made up 54% of all located individuals and were considered as representatives for the invertebrate community. Remaining species had by far smaller proportions and were not determined further due to the low individual numbers. Habitat preferences for the three dominant species were analyzed using negative binomial regression. Common and important habitat features were non-silted and coarse gravel areas, which are neighboured by patches with an elevation 1m above the water. Furthermore, the absence of vegetation cover as well the absence of ants was crucial for the occurrence of the three model species. Habitat preferences were subject to seasonal influences due to various requirements of different life stages. Other influencing factors were competition and predation due to Formicidae and larger individuals of Lycosidae. This demonstrates the high importance of structurally rich riverbeds with a mosaic of distinct habitat patches for the three representative species. Our findings are a valuable contribution for the conservation and management of braided rivers and their characteristic gravel bar biocoenosis.

## Introduction

### River landscapes as highly dynamic and harsh environments

River landscapes are constantly being disturbed, reshaped, destroyed and recreated by frequent current and flood pulses [1–7]. Alluvial habitat mosaics include all successional stages from bare gravel to tree-covered patches [8]. These dynamic changes can be found in particular in braided rivers, which are widespread in mountain valley areas in Central Europe [5]. At medium water levels, the main channel of a braided river divides into several side channels, which constantly divide and merge again. It includes gravel banks and islands that are subject to constant change due to dynamic processes such as erosion and accumulation due to lateral channel migration [6, 9–11]. Besides river dynamics, temperature is considered to be an important physical driver [12, 13] affecting habitat conditions and floodplain communities. Due to the lack of vegetation on gravel banks, exposed gravel can reach higher mean and daily pulse temperatures [13] than the air 1.5 meters above the ground. On sunny days, this difference can be up to 10 degrees [14]. Low water situations alternating with high discharge pulses in combination with high temperature fluctuations make floodplain areas and especially gravel banks highly dynamic and harsh environments.

### River landscapes serve as habitats for specialists

Over the course of evolution, organisms have evolved traits that allow them to survive in harsh environments. For example, such species have short and often asynchronous life cycles, high reproduction rates, and great dispersal capacities (r-strategists) [5, 15–18]. To cope with drought stress during low water levels, arthropods of sunny exposed habitats have for example a higher concentration of pigments, mostly melanin [19], to allow only a minimal amount of water to evaporate [20]. Iridescent and metallic colored wing cases seemed to be an advantage for sun and low humidity exposed coleopterans [19, 21, 22]. Furthermore, some carabid species have a dorsal-ventral flattened body which enables them to hide between the gravel [19, 21, 23]. Despite hot and dry conditions, riparian arthropods are adapted to frequent water level changes and flooding. These adaptations include, for example, the ability to move on the water surface, e.g. the two spider genera *Arctosa* and *Pardosa* make use of the surface tension [19, 24–26]. A hydrophobic hair structure in spiders also improves the chance of survival of individuals trapped in inundated gravel [27, 28]. Most riparian arthropods are also adapted to survive as eggs, larvae or adults in a submerged state for some period, depending on temperature and season [29–32]. The large ground beetle *Nebria picicornis* exhibits a spatial segregation of adults and larvae. The adults prefer the vicinity of the water line where food is more abundant, while the larvae live in higher areas that are less exposed to flooding [33]. Metapopulation formation is another way to minimize the impact of stochastic disturbances on the species’ survival at the landscape scale [27, 34].

A significant proportion of species living in these harsh environments are rare and often of conservation concern [12, 35–38]. Many of these species are already listed as endangered or even as threatened. To name but a few of them, two noticeable arthropod species living on gravel banks in river floodplains are the wolf spiders *Pardosa wagleri* and *Arctosa cinerea* (both Lycosidae, Araneae). Reasons for being listed as endangered are mainly habitat loss and fragmentation due to severe anthropogenic impacts in river floodplains. A changed flow regime can, for example, impede the creation of new bare gravel islands and bars while the old become overgrown, which leads to habitat loss, especially for pioneer species [5].

### River floodplains are among the most endangered landscapes worldwide

Nowadays, the large majority of rivers and floodplains in Europe are degraded. Specifically, only about 10% of the former floodplains in Germany are regarded to be in a near natural state [39–42]. The degradation of freshwater ecosystems increases habitat fragmentation and increases the risk of extinction for species in the remaining isolated pockets. In addition to pollution by nutrients and toxic substances, the hydrology and morphology of most rivers and river floodplains have been altered by straightening, channelization, damming or water withdrawal which consequently led to a significant reduction of aquatic-terrestrial transition zones [39, 43–45].

Recently, the economic, ecological and social importance of rivers and their floodplains has gradually moved to the foreground of societal concern, and the number of river-floodplain restoration projects has increased substantially [46–48]. However, most of these restoration projects focus on the river channel itself and rather neglect the riparian zone [43]. Especially for braided rivers, only a few but detailed studies exist about the requirements of habitat specialists in floodplains and on gravel banks, respectively [14, 15, 19, 49–51]. Thus, even if river-floodplain restoration comes into focus today, we lack information on floodplain species habitat requirements to facilitate restoration planning and optimize management concepts.

### Objectives of the present study

Based on the backdrop explained above, the main aim of our study was to understand and characterize habitat requirements of arthropods on gravel bars in river floodplains. For this end we focus on three model species *Pardosa wagleri*, *Arctosa cinerea* (both *Lycosidae*, Araneae) and *Paederidus rubrothoracicus* (*Staphilindae*, Coleoptera). These species occur in relatively high abundances and are representatives for gravel dominated river floodplains [12, 14, 52–55]. Concerning their habitat requirements, it is already known that these species do not occur in the entire area of the river corridor but have a patchy distribution which is due to their specific adaptations to gravel banks. However, until today only a few studies exist about the preferred habitat patches of single species [14, 15, 19, 49–51]. These studies consider a low vegetation density and open gravel bars to be of high relevance for the occurrence of the three model species. Lambeets et al. [50] and Steinberger [51] mentioned elevated gravel bars as a habitat characteristic without giving a definition of shape and position. Kühnelt observed a preference for areas about 40 cm above the normal water level of *A*. *cinerea* [19]. Moreover, it can be assumed that different life stages, e.g. juveniles, adults, and cocoon carrying adults, have different habitat requirements, due to e.g. differences in feeding behaviors and competition between juveniles and adults [56, 57]. Furthermore, own observations revealed that ants (Formicidae) aggressively prey on spiders, and adult spiders of *A*. *cinerea* and *P*. *wagleri* feed on juveniles of the same species. In the latter case, the juveniles (spiderlings) might, as an anti-predator behavior, occupy different habitat patches with distinct habitat characteristics, than the adults. Whether this influences the occurrence of model species was also be evaluated in the present study.

In short, the main aims of our study are (1) to provide a broader view on the habitat requirements of typical braided river floodplain arthropods, (2) to test for the relevance of elevated gravel bars for the species occurrence (3) to evaluate whether different life stages have different habitat requirements and (4) to test whether the co-occurrence of predacious ant (Formicidae) limit the occurrence of model species in the river corridor.

With this study, we contribute to a better understanding of the relevance of habitat requirements for typical and endangered floodplain arthropods and give valuable hints for the conservation and management of rivers and their floodplains.

## Methods

### Study area

**Fig 1:**
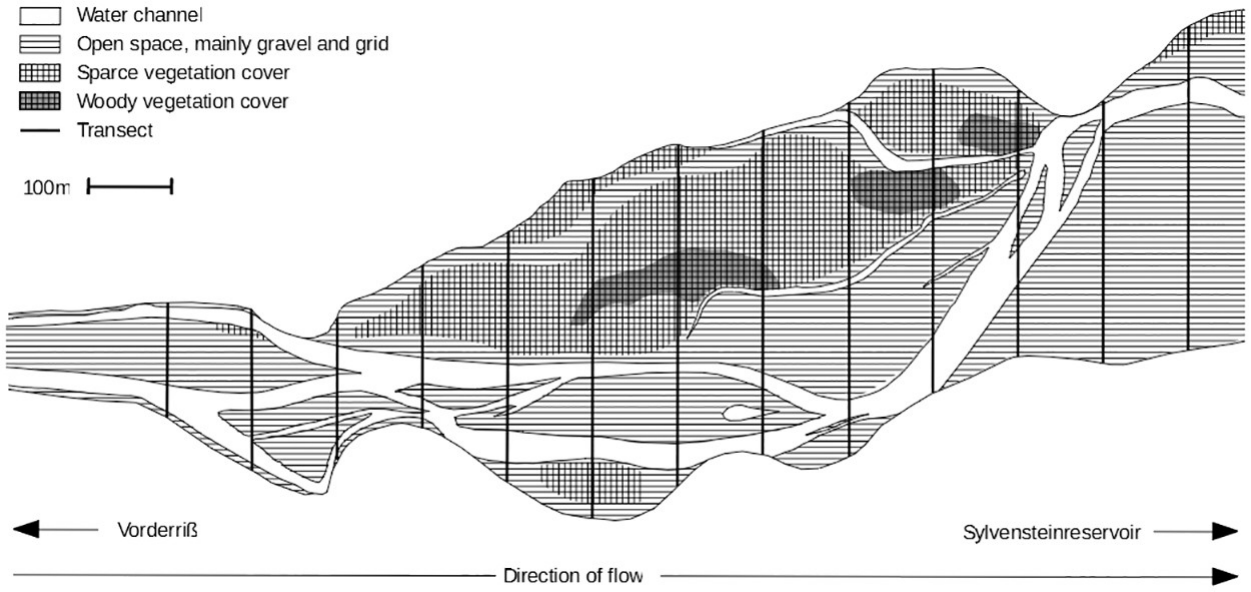
Map of the research stretch of the Upper Isar river, between the river-kilometer 233.2 and 234.4.

Studies were carried out in the Upper Isar river in Germany, upstream of the Sylvenstein Reservoir. This river stretch of the upper Isar is near-natural and highly dynamic, characterized by high rates of bed load transport despite of human influence. Frequently occurring flood events lead to the constantly changing formation of complex mosaics of habitat patches and their arthropod communities. Data were recorded at a representative 1200m long river stretch located between the river-kilometer 233.2 and 234.4 next to the small village Vorderriß. The selected river stretch was braided, consisted of multiple interweaving channels, and contained several differently sized islands and gravel bars either vegetation free or covered with, partly woody, dry vegetation in the uplifted areas (Fig 1). The river corridor was on average 190m wide (min: 70m, max: 240m).

### Data collection

Sample period lasted from May to July 2011. Within the selected river stretch, data on substrata and arthropod communities were recorded along 13 equidistant transects covering the river corridor, which was in our study defined as the aquatic/terrestrial transition zone between the left and right riverbank. On each transect, data were recorded on sample units with a size of 5m x 5m (compare [15]). The distance between sample units was 10m, which results in a number of 6 to 19 sample units per transect and a total number of 185 sample units.

For each sample unit 15 habitat variables were measured (Table 1). The height of the sample unit above water level was measured by a surveyor’s optical level. The vegetation density was estimated after the Braun-Blanquet-Skala [58] (Table 1). Substrates were classified according the AQEM field protocol [59] (Table 1). Moreover, the presence of steep slopes within a sample unit was recorded when sand and little stones were observed to trickle from the frail fringe. As tree covered island and riverbanks seems to have the same effect on the model species habitat choice, both parameters were combined for further data analysis.

**Table 1:** Recorded habitat variables on each of the 185 sample units. Minimum (Min), quartiles (QU), maximum (Max) and mean are given for all samples and each variable.

Habitat variables	Measurement unit	Min.	1^st^ Qu.	Median	Mean	3^rd^ Qu.	Max.	Number of sample units that fall into this category	Further description
Vegetation density	Density categories after Brown-Blanquet	0	0	0	4	6	75	71	
	0%	-	-	-	-	-	-	114	
	1%	-	-	-	-	-	-	11	
	6%	-	-	-	-	-	-	35	
	12%	-	-	-	-	-	-	16	
	20%	-	-	-	-	-	-	2	
	25%	-	-	-	-	-	-	3	
	50%	-	-	-	-	-	-	1	
	75%	-	-	-	-	-	-	3	
	100%	-	-	-	-	-	-	0	
Unstable steep slope	presence/absence	0	-	-	-	-	1	9	
Grain size of the substrate									
Siltation	in steps of 10%	0	0	30	34	70	100	103	
Soil and sand	Coverage in steps of 5%	0	0	0	8.4	5	100	49	< 0.2cm
Akal	Coverage in steps of 5%	0	0	0	4.2	0	100	28	0.2cm to 2cm
Microlithal	Coverage in steps of 5%	0	0	0	24.3	50	100	92	2cm to 6cm
Mesolithal	Coverage in steps of 5%	0	0	40	41.3	70	100	138	6cm to 20cm with a variable percentage of gravel and sand
Macrolithal	Coverage in steps of 5%	0	0	0	21.2	30	100	75	20cm to 40cm with a variable percentages of cobble, gravel and sand
Megalithal	Coverage in steps of 5%	0	0	0	0.7	0	90	6	> 40cm
Formicidae (ants)	individual number	0	0	1	5	5	50	96	
Height above water level	cm	0	39	74	79	113	300	all sample units	
Distance to elevation >1m	in steps of 25m	25	25	25	40	25	300	all sample units	
Distance to the next water channel	m	0	0	15	31	45	180	all sample units	
Distance to riverbank and woody vegetation cover	m	0	10	30	36	60	110	all sample units	
Bed-width	m³/s/m	36	38	39	49	48	128	all sample units	

The parameter “Distance to elevation > 1m”described the distance from the sample unit to the next gravel bar with an elevation of more than 1m above the water line, which remained unflooded by regular little summer-floods [60]. Water level rose partly 20 to 30cm over base flow condition for only a few times after thunderstorms and once up to 85cm over base flow condition during the investigation period. In case that sample units were located on islands within the water course, the distance to the next higher elevated gravel bar was set artificially high (2.000m) to express the limited reachability by non-flying arthropod species. As ants (Formicidae) might interact with the selected model species, the number of ants per sample unit was counted when there were ≤ 20 specimens. Numbers of > 20 species per sample unit were estimated in steps of five.

### Sampling of arthropods

Each sample unit was sampled once and was manually scanned for arthropods. Stones were turned over and finer substrates were raked with fingers. Besides the three model species, other riparian and terrestrial spiders, beetles and ants were sampled as well and abundance per sample unit was noted. Body sizes of *A*. *cinerea* and *P*. *wagleri* specimens were measured. Individuals with a body length ≤ 4mm were grouped as spiderlings and individuals ≥ 7mm/ ≥ 12mm (*P*. *wagleri*/*A*. *cinerea*) were grouped as adults. Medium sized individuals were assigned neither to spiderlings nor to adults. It was noted when adult spiders were carrying a cocoon. During wet or foggy weather conditions, most of the gravel bar inhabitants seek refuge inside the interstitial, therefore sampling was temporarily suspended during this time.

### Statistical analyses

To examine the spatial autocorrelation of species abundances on sample plots, the Moran’s I autocorrelation coefficient from the package “ape” was calculated [61]. With p > 0.001 a spatial autocorrelation between the sample units can be excluded.

**Fig 2:**
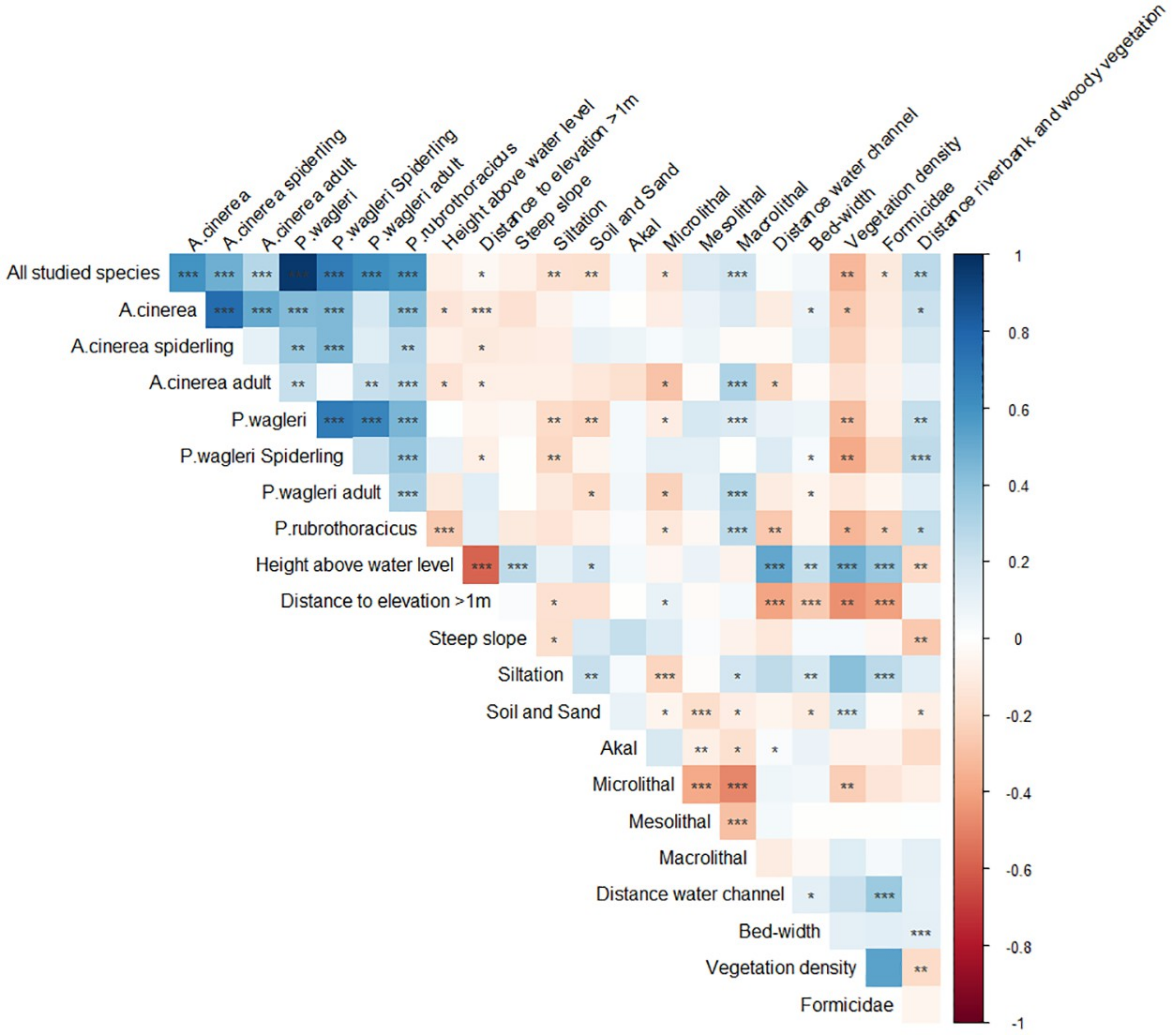
Correlation matrix after Spearman’s rank correlation (rho) and significant level stars. Color intensity of the glyph is proportional to the correlation coefficients.

The habitat variables (Table 1) were tested for collinearity (Fig 2) by the Spearman’s rank correlation test (rho). The abundance data of all three model species showed a high aggregation and was tested for overdispersion and expressed as the variance-to-mean ratio. At variance-to-mean ratio ≥ 1 and a clumping parameter k < 1, the negative binomial regression is regarded as the appropriate function for data modelling [62]. Species abundances were modelled in dependence of the habitat variables. First, a so-called full model was calculated which includes all habitat variables. In a second step, we used an automated selection procedure to select only the significant variables; model optimization was realized with the function “dredge” from package “MuMIn” which performs automated model selection with ‘all possible’ combinations based on the second order Akaike’s Information Criterion [63], corrected for small sample sizes (AICc). From hereafter the resulting model is called “final” model. Results from models that were as good as the final model, so-called competing models that had a delta AICc < 2, were also noted. Within the progress of calculating the final model, a third model was calculated. That is the so-called null model which includes none of the habitat variables. *Analysis of variances* (ANOVA) were calculated to compare the results of the three models, e.g. full, null and final model [64, 65]. Results for the ANOVA were expected to reveal significant differences between the final model the null model but not between the final model and the full model [66]. As the species data was not normally distributed the Nagelkerke´s pseudo R-square was calculated for each model to quantify the variance of the response variable explained through the regression model. In contrast to other pseudo R-squares, the Nagelkerke´s pseudo R-square can reach a maximum value of one. Thus, an r-square of R^2^ = 0.2 can be interpreted as acceptable, of R^2^ = 0.4 as good and an R^2^ = 0.5 as excellent [67].

Due to approximately 20% zero-valued observations in the data set, the performance of the full model for the abundance data was compared with a zero inflated regression model [68]. The comparison of both model results with the function “vuong” from the package “pscl” [68] showed a preference of the negative binomial regression over the zero inflated regression.

To test for co-occurrence of different species and the relationship between species occurrence and selected habitat variables the Spearman’s rank correlation (rho) was calculated [65]. Finally, the residuals of the deviance values were plotted to check for the required normal distribution to accept the selected model [65].

Spiderlings occurred in considerable numbers in the second half of the sampling period (from 27^th^ of June until the end of the sampling period) wherefore habitat analysis for spiderlings and adults were run separately with samples taken in this sample period (100 out of 185 sample units). These samples were subsequently subdivided in samples with (38) and without adults (62) to test for possible influence of adult individuals on the habitat choice of spiderlings.

All analyses were performed using “R” version 4.1.2 [69] with the packages “MASS” Version 7.3–45 [70], “fsmb” Version 0.5.2 [71] and “MuMIn” V*ersion 1*.*15*.*6* [72].

## Results

Altogether 2,642 individuals of riparian and terrestrial spiders, beetles and ants were caught. Araneae (spiders) with 1,422 individuals and a proportion of 51% of the total number of individuals made up the major taxonomical order, followed by the Formicidae (ants) with 978 individuals, Staphylinidae (rove beetles) with 154 individuals, and Carabidae (ground beetles) with 78 individuals. The dominant riparian species was *P*. *wagleri* with 1,092 individuals, followed by *A*. *cinerea* with 184 and *P*. *rubrothoracicus* with 154 individuals. Together, the three species account for 54% of the total number of individuals. Formicidae were dominated by *Formica selysi* (Formicidae). However, as the determination of all Formicidae at species level was not the focus of the present study, only information at the family level (Formicidae) was included in the models.

### Pooled species approach

**Fig 3:**
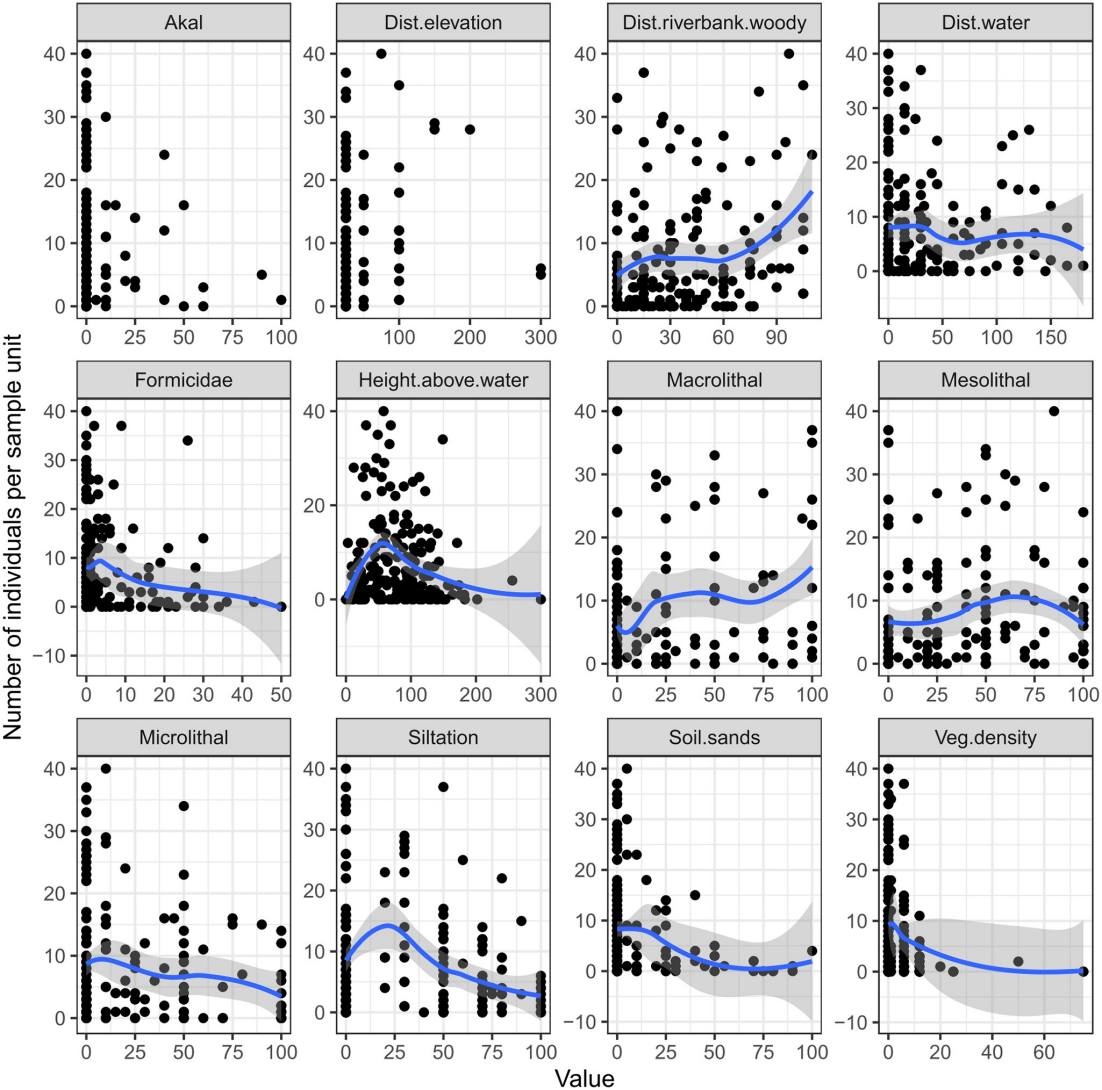
Number of pooled individuals of *P*. *wagleri*, *A*. *cinerea* and *P*. *rubrothoracicus* per sample unit plotted against 12 different habitat variables. The blue line represents the results of the locally weighted smoothing.

The number of individuals of *A*. *cinerea*, *P*. *wagleri* and *P*. *rubrothoracicus* on the sample units were checked for correlation. Here, significant correlations were found (Spearman’s rank correlation for *A*. *cinerea*–*P*. *wagleri*: rho = 0.44, p < 0.001; *A*. *cinerea*–*P*. *rubrothoracicus*: rho = 0.41, p < 0.001; *P*. *wagleri*–*P*. *rubrothoracicus*: rho = 0.45, p < 0.001). Therefore, as a first step, abundance data of the three model species *A*. *cinerea*, *P*. *wagleri* and *P*. *rubrothoracicus* was pooled for each sample unit to provide information on the habitat requirements of the invertebrate community living on the gravel banks of the braided rivers. The pooled abundances were plotted in dependence of the habitat variable (Fig 3) For information purposes, the corresponding scatterplots for the individual species can be found in the S1 Fig. The mean number of specimens on sample units decreased with increasing distance to the waterline. However, species abundances of all three species were comparably high on six sample units within a distance of 100 to 140m to the water line. These six sample units were located on plateau edges with unsilted gravel and sparse vegetation cover beside temporary water channels. The sampling of these six sample units took place 4 to 14 days after the temporary water channel was fed with water by a flood.

Moreover, it was checked whether the pooled number of model species correlates with the distance to the next riverbank or to the next available bank including riverbank and islandbank. Indeed, both were positively correlated (Spearman’s Rank correlation, rho = 0.267, p < 0.001 and rho = 0.262, p < 0.001).

Abundance data for pooled species showed a variance-to-mean ratio ≥ 1 (*A*. *cinerea* + *P*. *wagleri* + *P*. *rubrothoracicus* = 10.64) and a clumping parameter k = 0.802, wherefore a negative binomial regression was selected for modelling species abundance data [62] (hereafter referred to as “pooled species” model). All habitat variables were regarded to be independent as pairwise correlation coefficients among habitat variables were not higher than rho = 0.7 [73]. The pooled species approach revealed a final model with six variables influencing habitat suitability (R^2^ = 0.46; Table 2).

**Table 2:** Results of the final models for the pooled species approach, the three model species and for ants (Formicidae). Models for the two spider species were calculated based on all samplesand on samples taken in the second half of the sampling period. The numbers represents the z-value (estimate/ standard error) for each variable given by the dredged negative binomial regression. Significant results are given in bold. Percentages represent the relative proportion of occurrences of the variable in competing models with delta AICc < 2) [63].

	Pooled species	P. wagleri				A. cinerea			P. rubrothoracicus	Formicidae
	Entire time	Entire time		During spiderling glut		Entire time	During spiderling glut		Entire time	Entire time
		All	Cocoon	Spiderling	Adult	All	Spiderling	Adults		
Ind. Number	1430	1092	72	377	91	184	73	17	154	978
Distance to elevation >1m	**-3.570**	30%		-1.759	-1.629	**5.501**	**-2.005**		**-3.897**	**-3.546**
Vegetation density	**-4.226**	**-4.386**	17%	**-3.418**	33%	**2.773**	**-2.703**	17%	**-2.042**	**3.705**
Siltation	**-2.875**	**-2.464**		**-2.442**	**-2.224**	18%		-1.789	-1.554	**2.370**
Macrolithal	**5.149**	**2.452**	**5.273**	30%	**3.703**	5%		**2.884**	**2.305**	24%
Mesolithal	**2.987**	21%	**3.102**		**2.216**			1.705	22%	5%
Formicidae (ants)	**-2.283**	20%	**-2.895**	10%	10%	-1.403	5%	-1.368	63%	
Height above water level		**1.972**	100%		33%	-1.890	52%	30%	**-2.506**	**2.323**
Distance to riverbank and woody vegetation cover	17%	1.821	-1.864	**2.566**	33%		19%	9%	**2.122**	-1.815
Distance to the next water channel	17%	1.558	**2.430**		5%	36%	10%		**-2.360**	1.833
Soil and sand	17%	**-3.132**	38%			27%	**2.751**		6%	-1.881
Unstable steep slope	17%				48%	0.000	0.000	4%	53%	19%
Microlithal	17%	**-3.293**	17%	20%	14%	-1.871	-1.941	17%	25%	14%
Akal	17%		4%	20%				22%	3%	
Bed-width			-1.625	10%		5%	1.778		**-2.328**	10%
ANOVA (p-value) (Null- to final model)	< 0.001	< 0.001	< 0.001	< 0.001	< 0.001	< 0.001	< 0.001	-	< 0.001	< 0.001
ANOVA (p-value) (Full- to final model)	0.846	0.566	0.815	0.459	0.427	0.751	0.858	0.876	0.509	0.873
Nagelkerkes R^2^	0.46	0.47	0.55	0.57	0.38	0.50	0.39	0.53	0.5	0.43
Residual deviance (full model)	211.4	202.6	80.4	105.7	72.2	137.4	66.9	33.5	143.6	170.5
Residual degree of freedom (full model)	170	170	170	85	85	170	85	85	170	171
AICc (full model)	1098	1000	242	456	251	445	234	102	415	836
AICc (final model)	1083	991	228	440	235	432	217	81	406	824

One of the significant variables were elevated areas over 1m (Table 2). Significantly more individuals were found on gravel bars with access to elevations greater than 1 m than areas without this access (Wilcox rank-sum test, p = 0.0047). These elevated areas over 1m were found on transects with a discharge less than 1m³/s per meter riverbed-width.

Therefore, gravel islands with an elevation between 40 to 88cm had 4.4 more individuals of pooled species compared to gravel islands lower than 40cm, which were periodic submerged in water by small floods (Wilcox rank-sum test, p = 0.0022). In general, there were fewer individuals on islands than on gravel bars with a direct connection to the river bank (Wilcoxon rank sum test: p = 0.022).

### Species specific approach

#### Paederidus rubrothoracicus

Mean individual numbers were 0.8 individuals per sample unit. Maximum values were 10 individuals per sample unit. The sampling of this species took place over the whole sampling period without any peaks.

#### Pardosa wagleri

Mean individual numbers were 5.9 individuals per sample unit. Maximum values were 37 individuals per sample unit. While adult *P*. *wagleri* appeared throughout the sample period, cocoon carrying individuals were also found throughout the period, but peaked in the second half of June. Spiderlings of *P*. *wagleri* (≥ 4mm) occurred in great numbers at the end of June and in July.

#### Arctosa cinerea

Mean individual numbers were 0.995 individuals per sample unit. Maximum values were 13 individuals per sample unit. Only one cocoon carrying individual was sighted. It was located on an elevated, vegetated and heavy silted area with comparatively many ants (> 50 individuals), inside a burrow under a stone, with the entrance closed by woven silk. Final model results for adult individuals (≥ 12mm) and spiderlings (≤ 4mm) differed in terms of the habitat variables which were included in the models (Table 2).

### Spiderling distribution on sample patches with and without adult spiders present

A comparison of models for habitat suitability between spiderlings (≤ 4mm) on sample units with and without adult spiders (≥ 7mm for *P*. *wagleri* and *A*. *cinerea*), revealed disparate model versions (Table 3). With adults around, more spiderlings were found on sample units with less suitable habitat structures. For example, the vegetation cover only has a negative effect on the choice of habitat in the absence of adult animals. The model for spiderlings of *A*. *cinerea* without adults around is to be understood as an indication due to the insufficient Nagelkerkes R^2^ of 0.34 and not quite evenly distributed residuals.

**Table 3:** Habitat preferences calculated for spiderlings on sample units with and without adult spiders (individuals ≥ 7mm). Numbers for the variables represent the z-value (estimate/ standard error) given by the dredged negative binomial regression. Significant results are given in bold. Percentages represent the relative proportion of occurrences of the variable in competing models with delta AICc < 2 [63].

	A. cinerea Spiderling		P. wagleri Spiderling	
	+ Adults	- Adults	+ Adults	- Adults
Ind. number	42	22	226	147
Distance to elevation >1m	-1.635	45%	24%	**-2.910**
Vegetation density		-1.219	36%	**-4.953**
Silted substrate	13%		**-2.697**	
Formicidae (ants)				**-3.614**
Height above water level	**-2.709**		44%	**3.683**
Distance to riverbank and woody vegetation cover		**2.234**	**1.961**	
Distance to the next water channel	20%	9%	4%	40%
Soil and sand	**2.076**	9%	28%	
Akal	40%			**2.201**
Unstable steep slope	20%	9%	-1.850	**-2.610**
Macrolithal		9%		
Mesolithal			4%	
Microlithal		27%		20%
Bed-width				**2.403**
ANOVA (p-value) (Null- to final model)	0.004	0.005	< 0.001	< 0.001
ANOVA (p-value) (Full- to final model)	0.890	0.865	0.682	0.240
Nagelkerkes R^2^	0.42	0.34	0.46	0.86
Residual deviance (full model)	29.6	37.9	42.4	66.6
Residual degree of freedom (full model)	24	44	24	44
AICc (full model)	142	119	245	225
AICc (final model)	107	91	213	211

### Formicidae (ants)

The occurrence of ants was positively correlated with silted substrates and vegetation cover (Spearman Rank Correlation, rho = 0.27, p < 0.001 and rho = 0.53, p < 0.001). Silted substrates and vegetation cover were also positively correlated (rho = 0.42, p > 0.001). In contrast to silted substrates (rho = 0.10, p = 0.189), vegetation cover (rho = 0.47, p < 0.001) and the occurrence of ants (rho = 0.37, p < 0.001) were correlated with increasing elevations of gravel bars.

## Discussion

### Habitat requirements for braided river floodplain arthropods on gravel bars

Model results revealed the high relevance of gravel bars for the occurrence of the pooled species model, which mainly consisted of macro- and mesolithal, specifically head- to fist-sized cobbles. This finding is in line with other studies which focus on typical riparian arthropods in river floodplains [15, 19, 50, 51, 55, 74, 75]. Next to the substrate size, our study also clearly revealed the high importance of gravel bars to be open with interstitial spaces and not silted by fine sediments. We can only speculate on the reasons why gravel bars need to be open and with interstitial spaces. This space might serve as shelter, refuge during heat, bad weather conditions or flooding but also as living and hunting space. Therefore, first sampling trials for this study showed hardly any individuals at the surface of the gravel bars during rainfall. The occurrence of collembola in the sediments [19, 76] speaks for a use as hunting ground. Langhans and Tockner [35] counted 308 beetle species, of which more than one third occurred on the sediment surface as well as in the subsurface sediment. There is also a consensus in the literature about the negative effect of vegetation cover to riparian arthropods [14, 15, 19, 50, 55, 77] as observed in this study. The described link to the water channel [15, 19, 50, 51, 55, 78, 79] was subjectively confirmed due to many observed individuals next to the main channel. Occurrence of adult individuals of *A*. *cinerea* and *P*. *rubrothoracicus* were positive correlated with a nearby water channel. But in combination with other habitat parameters, the distance to a water channel was only considered in the final model for *P*. *rubrothoracicus* and with a negative impact on cocoon bearing *Pardosa wagleri* and Formicidae.

### Elevated areas as refuges

The parameter “elevated areas over 1m” had a significant influence for describing habitat suitability in this study. The reason for this might lie in repeated flood events. During smaller and more frequent floods more elevated instream gravel bars might be important as refuges. Rising water level of 20 to 30cm over normal water level was expressed by the focus of model species occurrence at a height between 40–60cm over normal water level (Fig 2) and by the small individual numbers on flat gravel islands lower than 40cm. Once water rose up to 85cm over normal water level. About once a year floods reached gravel bars over 100cm [60], last time in the previous year. If parts of higher gravel bars were just overflown by floodwater, without their relocation, individuals of the riparian invertebrate community could survive in the interstitial of overflown gravel bars [14, 38]. Bellmann [80] assumed *A*. *cinerea* to outlast flooding submerged in their burrow which was as well observed for a cocoon carrying female of *Arctosa maculata* during data acquisition to this study. Dondale and Redner [81] also observed females with cocoons in a burrow. However a single female of *A*. *cinerea* was found on an elevated, vegetated gravel bar, sitting with a cocoon in a hole under a stone, the entrance closed with silk. This was the only female of *A*. *cinerea* with a cocoon, despite many females with spiderlings on their back were found.

Nevertheless, the studied species avoided slopes with an angle more than 28°–29° because these become unstable due to the round and even grained gravel [82]. Especially drying sections, freshly carved by a flood, are affected by slippage when capillary cohesion weakens [83].

Due to the ability to move over greater distances [19, 49, 84], riparian arthropods were able to recolonize lower or new accumulated gravel bars after flooding immediately. Some carabids show increased dispersal activity after floods with traveled distances up to 280m in a couple of days [85, 86]. So, a gravel bar could be recolonized within a month [19]. Source for recolonization might be downstream drifting individuals, too. This thought is confirmed by a single marked *A*. *cinerea* which was found 2km downstream on an isolated gravel bank during a capture-recapture study [15]. Another ability to spread is moving on the water surface, which was observed for all three studied species during this sample period. Some of them were observed to be caught by the current and drifted downstream.

But even if the occurrence of riparian arthropods depends on the height of the area above the water level during floods, they were found most often on less elevated sections between 40 and 100cm. This finding is in line with the observation made by Kühnelt [19] about the habitat of *A*. *cinerea*. The habitat selection of *A*. *cinerea* is regarded as a tradeoff between the need to forage near the waterline and a safe hideway in rapidly rising water levels, around 30–40cm on rainy days.

### Species distribution in gravel dominated river floodplains

In the literature, a dependency of the studied species on the presence of water channels is described [15, 19, 50, 51, 55, 78, 79]. One reason for this may be food availability. In two studies, the highest availability of food within a braided river was found at the water channels [78, 87]. Already at a distance of 20m from the water channels, only 2% of the amount of prey available within the first few meters next to the water layer are left [78]. In concordance with this result Langhans and Tockner [35] found the main occurrences of arthropods less than 20m away from the water channels. Other studies identified a primarily terrestrial food source [19, 26, 49]. Once a mixed food composition was determined for *A*. *cinerea* and *P*. *wagleri* with a share of up to 56% and up to 80% for *P*. *rubrothoracicus* of aquatic insects [88]. A more detailed study showed a diet consisting entirely of terrestrial prey for beetles and lycosid spiders collected more than 50m from the water channels. For individuals collected next to a water channel a diet of mostly aquatic insects was determined [88]. On a Tyrrhenian sandy shore, the diet of *A*. *cinerea* consisted purely of adult individuals of *Talitrus saltator*, which were linked to the wash margin [89]. This underlines the dietary adaptability of the species. The present study on an extensive study area has not been able to show any clear spatial distribution pattern over the long period of time that could be explained by the food preferences described above. This could be explained by precipitation induced high water levels which lead to small scale changes in habitat quality and availability and corresponding changes in food sources such as drifted and washed ashore benthic and terrestrial invertebrates. Besides, there are also seasonal fluctuation in food sources e. g. due to seasonal emergence of aquatic insects [79, 88]. Another factor is air temperature, which determines the amount of available winged insects [90]. In addition, the type of surrounding vegetation influenced the proportion of terrestrial, aquatic or airborne diet. Thus, overhanging trees and bushes were excellent food sources of terrestrial prey [79, 91, 92]. Relative narrow river stretches, partly bordered by forest and advanced succession, accordingly, provide a good terrestrial prey source [19, 49, 79] in contrast to wide unvegetated river sections.

For *P*. *rubrothoracicus* different habitat requirements were described in the literature like a restriction to the river shoreline [55, 93], unvegetated river banks with different sediments, mud bottoms of dried-up rivers and lakes, including saline lakes [77, 94, 95] and fine sediments [14]. An observation during this study was a fast and straight, parallel to the river moving individual on neighbouring grasslands at a distance from 200m to the riverbed. Despite this the present study found a main distribution near the water channels. Single individuals were found on sparse vegetated plateau edges with stranded organic drift which served as further food source next to a water channel.

A change in habitat requirements dependent on developmental stage has been observed for *A*. *cinerea*. Framenau et al. [15, 49] presents in his results a migration of *A*. *cinerea* from gravel bars next to the channels to more distant areas. There they built tubes for hibernation. Similar migration was observed for populations at a mediterranean coastline. Individuals were found in the dune slack area especially during winter-spring months while in summer and in autumn this species occurred in more seaward areas [94]. The study at hand took place over the summer month, hence such a migration was not observed.

### Species composition

In river stretches enriched with gravel, the number of animal taxa caught and the proportion of each taxa differed in the individual studies [14, 35, 36, 74, 76, 96, 97]. What they all had in common, however, was the relatively high proportion of the taxa Araneae, Carabidae, Staphylinidae and Formicidae, with only a few dominant species. Smit et al. [97] observed just four species of Araneae and Carabidae providing more than 70% of all individuals in streams of low mountain ranges in Germany [97]. *P*. *wagleri* belonged to one of the dominant species, which together with four other spider species made up 75% of the total catch of all adult spiders in a survey on 10 alpine rivers [98]. At the river Isar, the species dominated with 59% the whole spider community [18]. For the current study, *P*. *wagleri* had a share of 41%, while all three model species represented 54% of all captives.

The number of taxa caught varied depending on the month of observation. Therefore, Langhans and Tockner [76] most likely collected only two taxa in February, September and November and 13 taxa in April. The focus on the two spiders and the Staphylinidea as representatives in this study was due to the lack of notable findings of carabidae (1.5%), despite the widespread opinion in the literature of some Carabidae as gravel bar specialists. One reason for this could lie in the season of the study period of our study. Another study in the same river stretch performed with similar methods but from early summer till autumn, showed 66% of the captured individuals belonging to the Carabidae [14]. Paetzold et al. [88] described the main occurrence of lycosid spiders (amongst others *A*. *cinerea*) occurring in June and August, while Carabidae dominated in April and October, a time period not included in our study.

### Spiderlings live in different habitats when adults are present

With the presence of large adult spiders, the occurrence of smaller spiderlings shifted slightly into fine-grained substrate types. In here, spiderlings find more hiding-places in small-grained substrates due to their body size, where larger predators cannot follow. The different habitat type choice between adult and juvenile spiders could be a result of different prey preferences due to different body sizes. In this context, Wise [57] reports a correlation between body size and cannibalism. With increasing body size difference between adults and juveniles, the rate of cannibalism increases. He also identified cannibalism as a major factor for density regulation, especially when prey is scarce. Following these results, habitat choice of spiderlings seems to be no self-contained choice but rather a response to this cannibalism. Remarkable is the positive effect of an increasing riverbed-width in dependence of the other variables to the spiderling numbers of *P*. *wagleri*. It is to be expected that the increasing space in the riverbed resulting from the increasing bed-width reduces the possibility of a get-together between spiderlings and adult individuals.

### Occurrence of preying ants limits habitat suitability for riverine floodplain arthropods

Frequent bedload exchange in the active part of the floodplain prevents the establishment of vegetation and ant nests. However, as soon as even sparse vegetation emerged and ants were able to establish, the three model species were no longer detected. Hering [87] already pointed out the high efficiency of ants foraging in gravel dominated river floodplains. In doing so they are aggressive competitors to other inhabitants of the floodplain areas [99]. During this study an immediate killing of a passing by spider was observed. The few located individuals of other species in these locations were attacked by ants as soon as they were exposed during the research. This aggressive foraging behavior might be a result of the limited food supply of the habitat. Lude et al. [100] counted 0.4% prey carrying individuals of 9,539 returning individuals of *Formica selysi*. In this manner, ants can alter the distribution of other invertebrates like observed in this study. Hence, ants used as covariate by Jonsson et al. [101] did not change the results but explained a significant amount of the variance in the distribution among several terrestrial taxa. An influence on individual numbers due to ants was found by Manderbach and Reich [36]. Therefore, the Upper Isar downstream of the Sylvenstein-Reservoir showed fewer wide riverbeds with narrow, almost unchanged stable gravel bars which were visited by Formicidae. There individual numbers of Carabidae were six times lower than in wide riverbeds upstream of the Reservoir.

## Conclusions

This study clearly shows that the mere presence of gravel bars is not sufficient to harbor populations of the three species *Pardosa wagleri*, *Arctosa cinerea* and *Paederidus rubrothoracicus*.

Of importance are structurally rich riverbeds with a mosaic of distinct habitat patches for the three representative species. In this way different requirements of different circumstances due to predation, competition, developmental stage or environmental events, like floods or seasonal changes, are fulfilled. Structures of high relevance were elevated and open, not silted gravel bars with different substrates for the occurrence of the three representative species of river floodplain arthropods. All these structures are part of a natural, or semi-natural, free flowing braided river with continuously changing riverbed structure, belonging to a lateral migration of the river channels. To preserve, support and develop populations of the model species, rivers need space. These complex riverine landscapes represent unique ecosystems and habitats for many specialists, such as the three species we examined in our study. These ecosystems must be protected and preserved.

## Supporting information

S1 Table Raw data for each sample unit. (CSV)

S1 Fig Scatterplots for *Pardosa wagleri*, *Arctosa cinerea* and *Paederidus rubrothoracicus* in dependence of the habitat variables. (PDF)
