# Supplementary material for: Habitat requirements of riparian arthropods on gravel bars: Implications for conservation and management of braided river floodplains
Source: PLoS One. 2022 Sep 27;17(9):e0274977. doi: 10.1371/journal.pone.0274977 (PMC9514604; doi:10.1371/journal.pone.0274977)
Supplement: S1 Fig — (PDF) [file pone.0274977.s002.pdf]

Habitat requirements of riparian arthropods on gravel bars: Implications for conservation and management of braided river floodplains

Reena Wessels, Andrea Sundermann

Electronic appendix S2

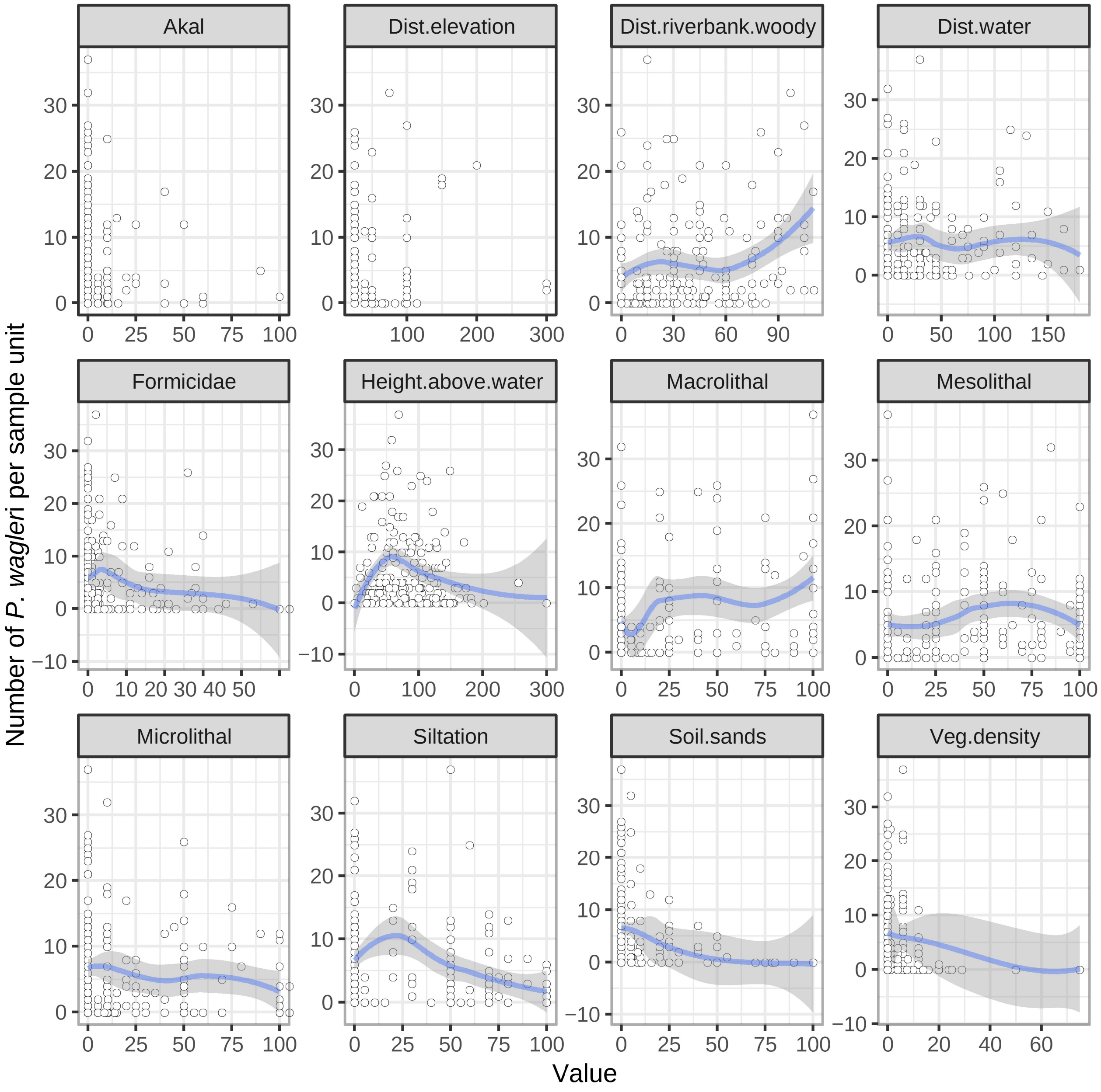

Individual number of *P. wagleri* per sample unit plotted against 12 different habitat variables. The blue line represents the results of the locally weighted smoothing.

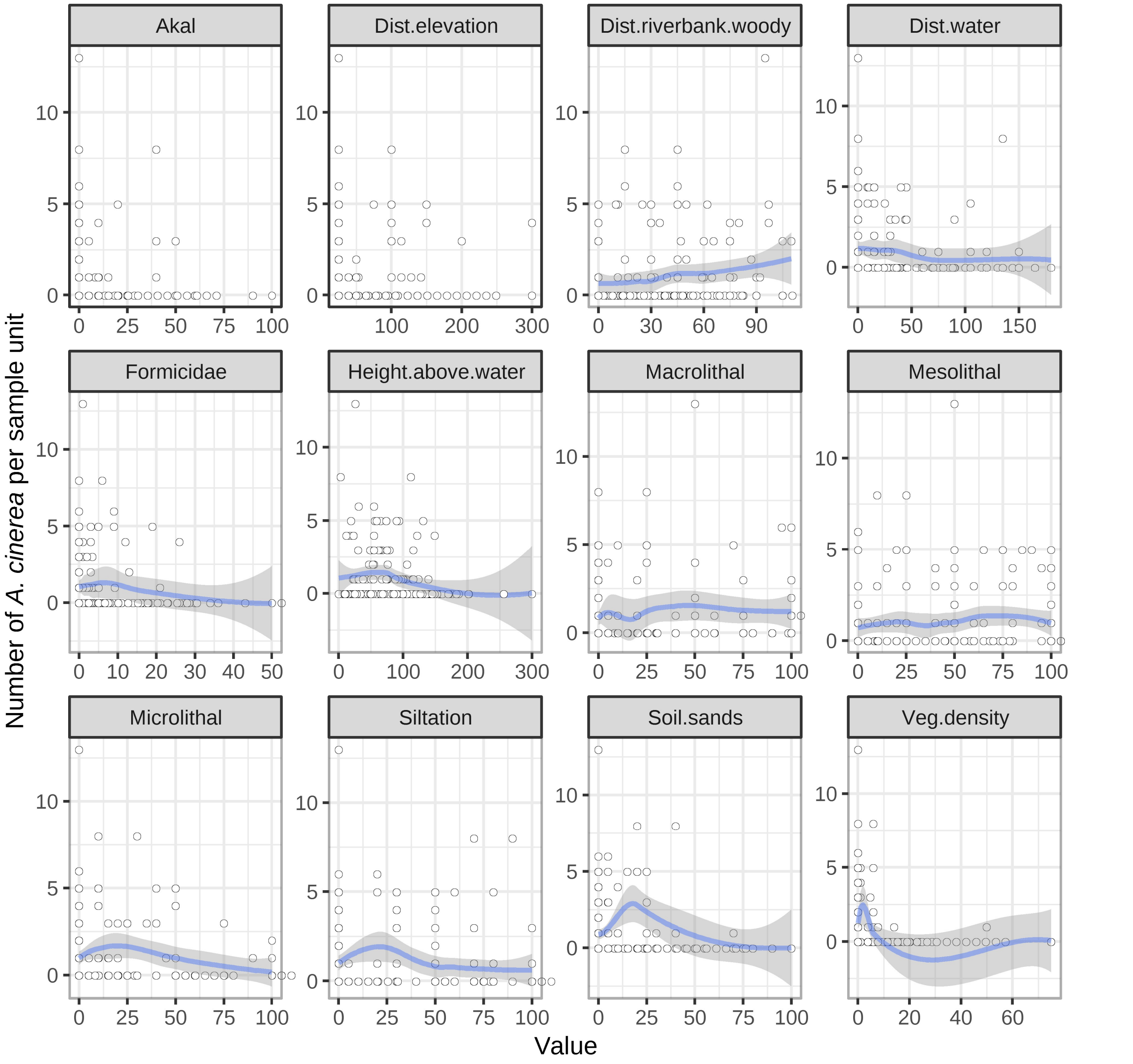

Individual number of *A. cinerea* per sample unit plotted against 12 different habitat variables. The blue line represents the results of the locally weighted smoothing.

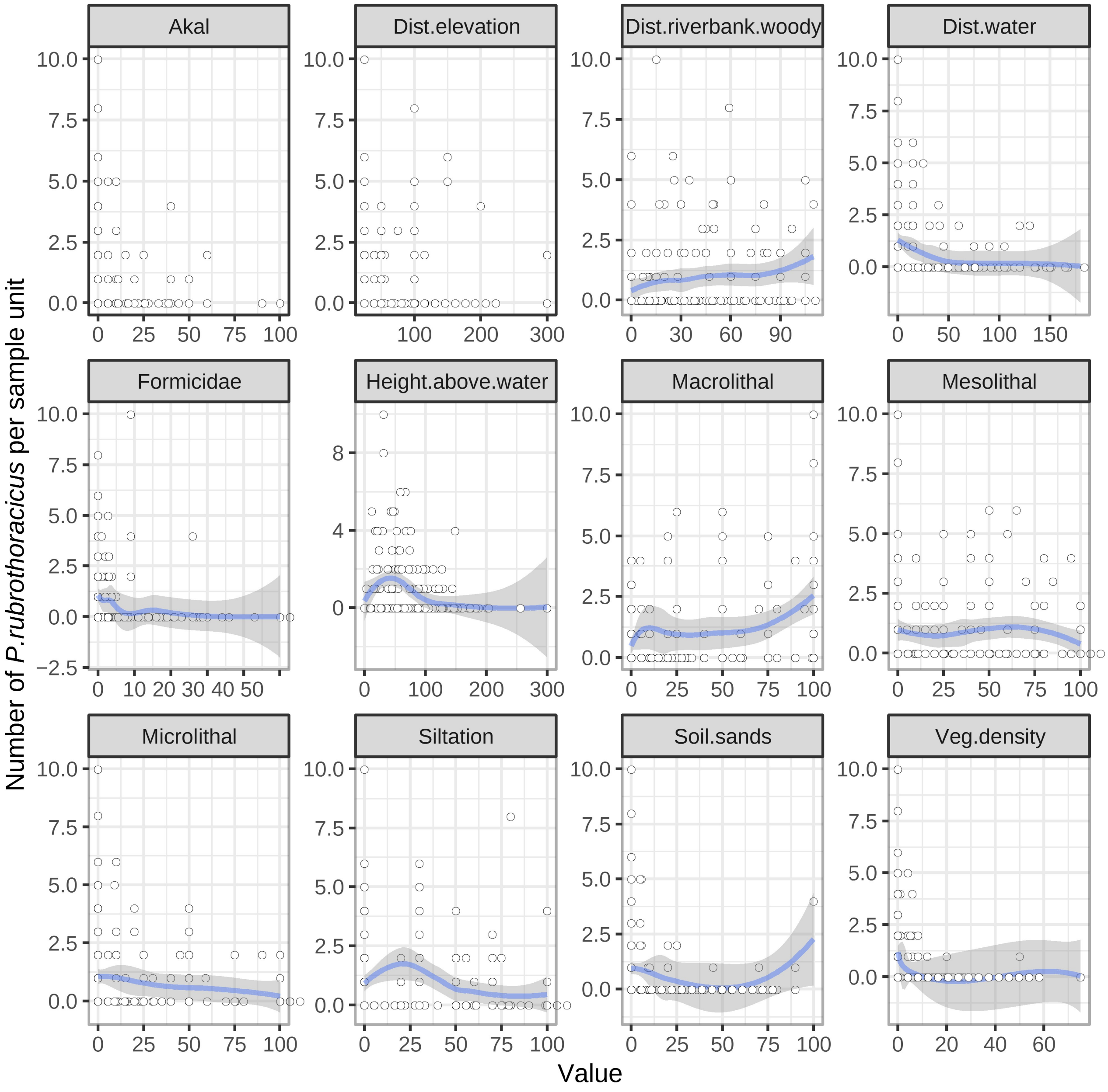

Individual number of P. rubrothoracicus per sample unit plotted against 12 different habitat variables. The blue line represents the results of the locally weighted smoothing.
